# Transparency, reproducibility, credibility: announcing a pilot

**DOI:** 10.1186/s12915-015-0175-8

**Published:** 2015-08-06

**Authors:** Penelope Austin, Miranda Robertson

**Affiliations:** BMC Biology, BioMed Central, 236 Gray’s Inn Road, London, WC1X 8HL UK

The central principle of open access is to make the results of research freely available to all, and as a pioneer of this model of scientific publishing, BioMed Central has always recognised that realising the full value of this accessibility depends on transparent reporting of the experimental work, as well as making the data available for others to analyze and re-use. This general principle is stated in our guide to authors, while referees who assess our research articles are asked to consider whether the methods are adequately reported, whether the data shown include the necessary controls and are adequate to support the conclusions drawn, and whether they have been able to assess the validity of the statistics. Authors and reviewers are also pointed to various internet-hosted reporting checklists relevant to different types of research.

There is arguably a need for more, however. In the wake of recent concern about the unreliability and irreproducibility of a significant proportion of published preclinical research [1, 2], the risk of a consequent loss of public trust, and the waste when such research is used as a basis for costly clinical trials, both the publishers and funders of biomedical research have recognised the need to impose reporting standards. One outcome has been the publication of principles and guidelines for reporting preclinical research by the NIH [3] along with a call for the use of checklists by endorsing journals, in a bid to raise consciousness and standards in this area, and facilitate the interpretation and repetition of published work.

From this month four Biomed Central journals — *BMC Biology*, *BMC Neuroscience*, *Genome Biology* and *GigaScience* — will pilot the use of a checklist for our submitting authors [4]. This short set of guidelines reflects four central requirements in accordance with BioMed Central’s general commitment to full and transparent reporting, and with the principles set out by the NIH. First is the need for transparency, so that, for example, the number of replicates is clearly stated and biological and technical replicates can be distinguished. Second, the importance of appropriate statistical analysis. Third, a requirement for the reagents used, such as antibodies, to be unambiguously identified. Fourth, that the data and materials on which the conclusions of published work rely should be made available.

The difficulties of attempting a set of requirements that are not too dogmatic or unduly burdensome and are appropriate to the wide range of research we publish should not be underestimated, and there are arguably dangers in constraining all research to standards best applied to established fields and well defined systems. In developing our checklist, we have benefited both from the experience of authors with the checklists of other journals and from the advice of our Editorial Board, a selection of whose comments can be found in our blog [5].

With the aim of not overburdening submitting authors whose papers meet the standards set, we do not ask for page and line numbers for each piece of information, but instead for confirmation that applicable requirements have been met, and/or for explanations where this has not been fully achieved. The checklist and authors’ answers in reference to it will play a part in our editorial assessment of whether a paper should be sent for peer review, and help avoid the situation where reviewers are hindered in their ability to assess a paper because information is missing. Reviewers will also see the authors’ answers and be able to advise us whether they are acceptable or not.

During the pilot phase for the checklist, we shall be collecting feedback and monitoring the effects on the transparency of submissions and the reports of our referees, with a view to striking a sensible balance between enforcing rigor and impeding progress.

Of course a checklist cannot itself guarantee reproducibility. By insisting on transparency it should make clear the degree to which the conclusions of each published study have been rigorously tested, but this does not rule out the possibility that subsequent studies may call these conclusions into question, and in such cases we recognize the obligation to publish a convincing rebuttal.
